# Effect of enteral diet enriched with eicosapentaenoic acid, gamma-linolenic acid, and antioxidants in patients with sepsis-induced acute respiratory distress syndrome

**DOI:** 10.1186/s40560-015-0087-2

**Published:** 2015-05-19

**Authors:** Kunihiro Shirai, Shozo Yoshida, Naoki Matsumaru, Izumi Toyoda, Shinji Ogura

**Affiliations:** Department of Emergency and Critical Care Medicine Ichinomiya Municipal Hospital, Bunkyo, Ichinomiya, Aichi 491-8558 Japan; Department of Emergency and Disaster Medicine, Gifu University Graduate School of Medicine, Gifu, Japan

**Keywords:** Acute respiratory distress syndrome, Antioxidants, Eicosapentaenoic acid, Enteral nutrition, Gamma-linolenic acid, Sepsis

## Abstract

**Background:**

In this study, the effects of an enteral diet enriched with eicosapentaenoic acid (EPA), γ-linolenic acid (GLA), and antioxidants were compared with a standard enteral diet in critically ill patients with sepsis-induced acute respiratory distress syndrome (ARDS).

**Methods:**

This study was a single-center, prospective, randomized, single-blind, controlled trial in our Advanced Critical Care Center. Patients were randomized to receive a continuous EPA, GLA, and antioxidant-enriched diet (study group), or an isocaloric standard diet (control group).

**Results:**

Twenty-three of 46 patients were in the study group, and the other 23 were in the control group. Duration of mechanical ventilation, incidence of new nosocomial infections, changes over time in Sequential Organ Failure Assessment (SOFA) scores, and 60-day mortality were not significantly different between the two groups. The ratio of partial pressure of oxygen to fraction of inspired oxygen on day 7 was significantly higher in the study group (233.0 [185.5–282.8] vs. 274.0 [225.5–310.8], *p* = 0.021). Duration of ICU stay was significantly shorter in the study group than in the control group (24.0 [20.0–30.0] vs. 15.0 [11.0–24.0], *p* = 0.008).

**Conclusions:**

An enteral diet enriched with EPA, GLA, and antioxidants did not improve duration of mechanical ventilation, SOFA score, incidence of new nosocomial infections, or mortality but did favorably influence duration of ICU stay in critically ill patients with sepsis-induced ARDS.

**Electronic supplementary material:**

The online version of this article (doi:10.1186/s40560-015-0087-2) contains supplementary material, which is available to authorized users.

## Background

Acute onset of respiratory failure with protein-rich pulmonary edema attributable to increased permeability of alveolar epithelium and endothelial injury in pulmonary vessels is the characteristic features of acute respiratory distress syndrome (ARDS) [1].

Researchers have identified many of the inflammatory mediators involved in the pathogenesis of ARDS and sepsis, including reactive oxygen species and lipid mediators (e.g., eicosanoids) [2,3]. Eicosanoids play a role in both pro- and anti-inflammatory reactions, depending on the original fatty acids [4]. Pro-inflammatory eicosanoids originate from n-6 fatty acids such as arachidonic acid (AA). Competing with AA are eicosapentaenoic acid (EPA) and docosahexaenoic acid (DHA), which originate from n-3 fatty acids and produce eicosanoids that are less markedly inflammatory and even anti-inflammatory [5]. Supplementation with n-3 fatty acids (fish oil) reportedly improves clinical outcomes in critically ill patients [4,6]. Furthermore, γ-linolenic acid (GLA) and dihomo-GLA supplements reduce synthesis of pro-inflammatory AA metabolites [7]. Additionally, strong promotion of oxidation occurs during development of sepsis and ARDS [8]. Supplementation with antioxidants such as selenium, zinc, vitamin E, and vitamin C improves antioxidant capacity, and thus outcomes, in critically ill patients [9]. Choice of nutritional supplement is particularly crucial in sepsis-induced ARDS because the lungs are the major sites of production of eicosanoids [10].

Three randomized controlled studies have reported clinical benefits from administering enteral nutrition enriched with EPA, GLA, and antioxidants in patients with ARDS [11], acute lung injuries [12], severe sepsis, and septic shock [13]. A meta-analysis showed that these nutritional supplements reduce mortality, secondary infection, and length of hospital stay [14]. However, these studies may be biased in favor of the study groups because they received n-3 fatty acids, which are metabolized into weakly inflammatory eicosanoids, whereas the control groups received n-6 fatty acids, which are metabolized into more strongly inflammatory eicosanoids. Furthermore, the patient groups differed in their proportions of non-sepsis and sepsis-related ARDS, which is important because these subgroups have different prognoses [15-17]. Thus, the purpose of our study was to compare the effects and safety of enteral diets enriched with EPA, GLA, and antioxidants vs. standard isocaloric and isonitrogenous diets in critically ill patients with sepsis-induced ARDS.

## Methods

### Study design

This study was a single-center, prospective, randomized, single-blind, controlled trial conducted between March 2008 and March 2010 in the Advanced Critical Care Center, Gifu University Hospital. The study protocol and consent forms were approved by the institutional review boards of Gifu University Hospital. Written informed consent was obtained from all patients or their legal representatives before inclusion in the study.

### Study patients

Eligibility criteria were ages more than 18 years, receiving mechanical ventilation and able to be fed enterally within 24 h of diagnosis of sepsis-induced ARDS. Diagnoses of severe sepsis and septic shock were made according to the criteria defined by the American College of Chest Physicians/Society of Critical Care Medicine Consensus Conference Committee [18]. ARDS was diagnosed according to the criteria defined by the American-European Consensus Conference [19]. Multiple organ dysfunction syndromes were evaluated by total Sequential Organ Failure Assessment (SOFA) scores [20]. After inclusion, eligible patients were randomly assigned by a principal investigator in a 1:1 ratio to either study or control groups. For our exclusion criteria, please see Additional file 1.

### Study protocol

Eligible patients were randomly allocated to two groups: a study group that received an enteral diet enriched with EPA, GLA, and antioxidants (Oxepa™; Abbott Nutrition, Tokyo, Japan) and a control group that received a standard isocaloric enteral diet (Ensure Liquid™; Abbott Nutrition, Tokyo, Japan). The compositions of these diets are summarized in Additional file 1. The study diet, a high-fat, low-carbohydrate enteral formulation contained more calories per 100 mL than the control diet. The control diet did not contain EPA or GLA, and several antioxidants, such as vitamins E and C, were in lower concentrations than in the study diet. There were more carbohydrates in the control diet than in the study diet.

The goal calories for both groups were set at 1.2 × basal energy expenditure (BEE) calculated using the Harris Benedict equation and adjusted to ideal body weight. The diets delivered 70% of goal calories in the first week and 100% within 14 days.

The day the enteral diets began was set as day 1. Gastric or jejunal tubes were inserted as determined by the principal investigator, with jejunal tubes being inserted under fluoroscopic guidance. The diets were administered continuously by infusion pumps starting within 24 h of diagnosis of sepsis-induced ARDS. Enteral administration was discontinued when oral intake became possible or when the principal investigator decided to discontinue them because of diet-related adverse events. Severe sepsis, septic shock, and ARDS were treated in accordance with the Surviving Sepsis Campaign Guidelines and ARDS Network Low Tidal Volume Ventilation Protocol [21,22]. This accordance includes our extubation process. We confirm that this study has no influence on the decision of discharge from ICU.

### Data collection

The following data were collected for all patients: age, sex, diagnostic category (medical, surgical, or trauma), baseline comorbidities, Acute Physiology and Chronic Health Evaluation (APACHE) II and total SOFA scores on diagnosis, number of organ failures, BMI, height, actual and ideal body weights, 1.2 × BEE (goal calories), and calorie intake on days 7 and 14. Because the enteral diets supply relatively large amounts of fat, the following nutritional variables were assessed once a week (days 1, 7, and 14): total serum cholesterol, triglyceride, albumin, aspartate transaminase, alanine transaminase concentrations, and platelet count.

### Outcome measures

The primary end point of this study was the duration of mechanical ventilation. Secondary end points included duration of ICU stay, changes over time in total SOFA score, partial pressure of oxygen/fraction of inspired oxygen (PaO_2_/FiO_2_) ratio (assessed on days 1, 3, 5, 7, and 14), development of new nosocomial infections, and all-cause mortality within 60 days of diagnosis. For our weaning protocol and ICU discharge protocol, see Additional file 1.

### Statistical analysis

Data are expressed as percentages or medians with interquartile ranges. When comparing two values in parentheses linked with “vs.”, the former value is for the control and the latter is for the study group, unless otherwise noted. All *p* values are two tailed; *p* < 0.05 is considered statistically significant. All analyses were performed on an intention-to-treat basis using IBM SPSS statistics version 19 (IBM, Somers, NY, USA). The Mann-Whitney *U* test was used for continuous variables and Fisher’s exact test for categorical variables. The intragroup proportions were analyzed using a *z*-test with the Bonferroni adjustment. To construct survival curves showing time to discontinuation of ventilator support and time to discharge from the Advanced Critical Care Center, the Kaplan-Meier survival analysis method with the log-rank (Mantel-Cox) test was employed.

## Results

**Figure 1:**
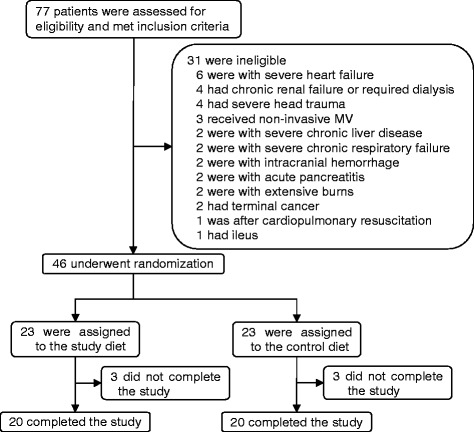
Patient flow across the study. MV, mechanical ventilation.

Forty-six patients were eligible for this study, 23 of whom received the study diet and the remainder received the control diet. No patients were withdrawn from the study, and the sole reason for the incomplete study was the death (Figure 1). Relevant patient variables were comparable between the two groups, as shown in Table 1. There were no significant differences in age, sex, reason for admission, and baseline comorbidities. The median APACHE II and SOFA scores were not significantly different, and the number of organ failures was similar in the two groups. There were no significant differences between the groups in infection sites, the most frequent being pulmonary (16 patients altogether, 34.8%). The median starting time for enteral nutrition was 20 h after meeting the inclusion criteria in both groups. Both groups had achieved their target calories by the seventh (70% of the goal calories) and fourteenth days (100% of the goal calories). There were no significant differences between groups in calorie or protein (51.0 g/day vs. 62.5 g/day) supplied by day 14. The pathogenic bacteria causing ARDS are listed in Table 2, and the definitive antibiotic therapy are shown in Table 3.

**Table 1:** **Relevant patient variables and baseline characteristics of patients enrolled in this study**

	**Study group (** ***n*** **= 23)**		**Control group (** ***n*** **= 23)**		***p*** **value**
Age, years	71 (66–77)		74 (60–80)		0.717
Gender, *n* (%)					0.314
Male	15 (65.2)		19 (82.6)		
Female	8 (34.8)		4 (17.4)		
Reason for admission, *n* (%)					0.418
Medical	5 (21.7)		6 (26.1)		NS
Elective surgery	6 (2.1)		4 (17.4)		NS
Trauma	4 (17.4)		9 (39.1)		NS
Emergency surgery	5 (21.7)		3 (13.0)		NS
Burn	3 (13.0)		1 (4.3)		NS
EN start time, hours	20 (8–24)		20 (8–24)		0.805
Catecholamine use, *n* (%)	16 (69.6)		14 (60.9)		0.758
Baseline comorbidities, *n* (%)					
Hypertension	12 (52.2)		14 (60.9)		0.767
Cardiac disease	11 (47.8)		7 (30.4)		0.365
Diabetes mellitus	7 (30.4)		5 (21.7)		0.738
Oncological disease	2 (8.7)		4 (17.4)		0.665
Chronic obstructive pulmonary disease	2 (8.7)		3 (13.0)		1.000
Chronic kidney disease	2 (8.7)		3 (13.0)		1.000
Cerebrovascular disease	2 (8.7)		3 (13.0)		1.000
Liver disease	1 (4.3)		1 (4.3)		1.000
APACHE II score on onset	24 (21–28)		23 (21–26)		0.860
SOFA score on onset	10 (6–13)		9 (8–13)		0.947
No. of organ failures, *n* (%)					0.274
1	8 (34.8)		6 (26.1)		NS
2	6 (26.1)		12 (52.2)		NS
3	7 (30.4)		3 (13.0)		NS
4	2 (8.7)		2 (8.7)		NS
Infection sites, *n* (%)					0.851
Pulmonary	7 (30.4)		9 (39.1)		NS
Bacteremia	4 (17.4)		6 (26.1)		NS
Abdomen	3 (13.0)		3 (13.0)		NS
Soft tissue	2 (8.7)		2 (8.7)		NS
Burn wound	3 (13.0)		1 (4.3)		NS
Urinary tract	3 (13.0)		1 (4.3)		NS
Others	1 (4.3)		1 (4.3)		NS
Tidal volume, mL/kg	7.1 (6.6–7.8)		7.0 (6.3–8.4)		
PEEP, cmH_2_O	11.0 (9.0–15.0)		11.0 (9.0–14.0)		
Plateau airway pressure, cmH_2_O	28.2 (23.6–30.6)		26.8 (21.6–31.5)		
Respiratory rate, breaths/min	23.0 (17.0–27.0)		22.0 (18.0–25.0)		
Ejection fraction, %	62.4 (43.7–69.3) (*n* = 21)		61.3 (45.0–78.5) (*n* = 19)		
Sivelestat sodium use, *n* (%)	21 (91.3)		19 (82.6)		
Steroid use, *n* (%)	11 (47.8)		12 (52.2)		
Sedation drugs					
Midazolam use, *n* (%)	19 (82.6)		21 (91.3)		
Propofol use, *n* (%)	3 (13.0)		2 (8.7)		
Dexmedetomidine use, *n* (%)	4 (17.4)		4 (17.4)		
Analgesics					
Fentanyl, *n* (%)	15 (65.2)		13 (56.5)		
Buprenorphine, *n* (%)	8 (34.8)		10 (43.5)		
Actual body weight, kg	60 (55–66)		63 (58–66)		0.409
Ideal body weight, kg	59.9 (54.2–62.1)		59.9 (56.3–60.6)		0.930
Body mass index	23.2 (21.3–23.7)		23.1 (22.0–23.9)		0.684
Goal calories, kcal/day	1453.6 (1347.9–1574.0)		1454.2 (1364.5–1569.6)		0.956
	Day 7	Day 14	Day 7	Day 14	
Calorie intake, kcal/kg/day^a^	18.78 (18.12–20.21)	24.22 (23.32–25.90)	19.48 (15.73–20.68)	24.32 (22.67–25.75)	
Protein intake, g/kg/day^b^	0.781 (0.700–0.837)	0.988 (0.933–1.063)	0.613 (0.529–0.683)	0.810 (0.749–0.863)	

*NS Not Significant, EN Enteral Nutrition, APACHE* Acute Physiology and Chronic Health Evaluation, *SOFA* Sequential Organ Failure Assessment, *PEEP* Positive end-expiratory pressure. ^a^The difference at day 7 or day 14 is not statistically significant with *p* = 0.965 and *p* = 0.818, respectively; ^b^The differences at day 7 and day 14 are both statistically significant (*p* < <0.001).

**Table 2:** **Pathogenic bacteria causing acute respiratory distress syndrome**

	**Study group**	**Control group**
Methicillin-sensitive Staphylococcus aureus	3	5
Methicillin-resistant Staphylococcus aureus	2	3
Streptococcus pneumoniae	1	0
Streptococcus pyogenes	0	1
Enterococcus faecalis	1	1
Escherichia coli (ESBL)	6 (1)	3 (1)
Klebsiella pneumoniae	1	3
Haemophilus influenzae	0	2
Pseudomonas aeruginosa	6	1
Enterobacter cloacae	2	3
Serratia marcescens	0	1
Acinetobacter baumannii	0	1
Citrobacter koseri	0	1
Stenotrophomonas maltophilia	1	0
Prevotella species	0	2
Bacteroides species	3	2
Polymicrobial infection	3	3

**Table 3:** **Definitive antibiotic therapy**

	**Study group**	**Control group**
Ampicillin	2	2
Sulbactam/Ampicillin	0	3
Piperacillin	3	3
Tazobactam/Piperacillin	2	4
Cefazolin	3	3
Cefotiam	2	1
Ceftriaxone	1	1
Ceftazidime	2	0
Meropenem	5	3
Vancomycin	2	3
Sulfamethoxazole/Trimetoprim	1	0

The numbers of cases in which patients were admitted due to septic ARDS were 16 in the study group and 10 in the control group. In passing, the standard diet was fed enterally to all of the registered patients developing septic ARDS during the ICU stay before starting the study. Seven cases in the study group (30.4%) were included because of septic ARDS developed during ICU stay. The duration from the ICU admission to the onset of septic ARDS was 8.0 days (median, IQR: 6.5–13.0) in the study group. In the control group, there were 13 cases (56.5%) with the duration of 5.0 days (median, IQR: 4.0-8.0). This difference of the ratio was insignificant according to the Fisher’s exact test (*p* = 0.136).

### Primary and secondary outcomes

**Figure 2:**
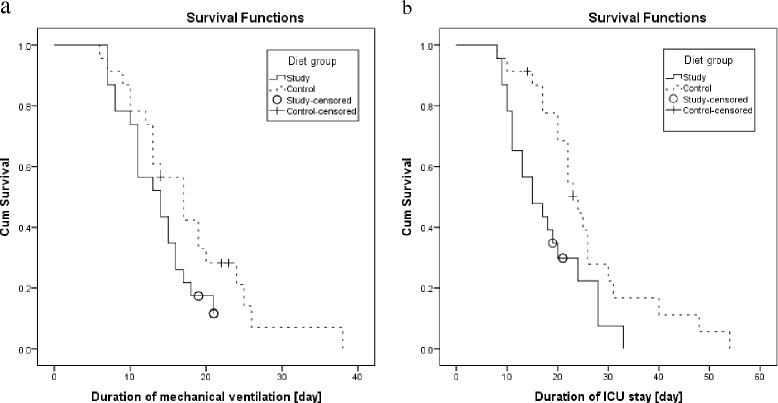
Survival curves generated by Kaplan-Meier survival analysis, representing primary and secondary clinical outcomes. **(a)** Duration of mechanical ventilation. **(b)** Duration of ICU stay. The sole reason for censoring was that three patients in each group died before completing the study (on days 14, 23, and 23 in the control group and on days 19, 21, and 21 in the study group). One patient in the control group died on day 23 but was extubated on day 22. Cum, cumulative.

Table 4 shows the clinical outcomes. Duration of ICU stay was the only outcome that significantly improved in the study group (24.0 [20.0–30.0] vs. 15.0 [11.0–24.0], *p* = 0.015) with a power of 35%. Ten patients (43.5%) in the study group had a relapse of infection, as did 12 patients (52.2%) in the control group. There were no significant differences between groups in incidence of new nosocomial infection or mortality (13.0% in both groups). The primary outcome, duration of mechanical ventilation, was shorter in the study than in the control group, but this difference was not significant (17.0 [12.0–24.0] vs. 14.0 [10.0–17.0], *p* = 0.115) with a power of 9.7%. Survival curves showing changes over time in number of patients requiring ventilator support (Figure 2a) and patients remaining in ICU (Figure 2b) are depicted in Figure 2. In both graphs, the lines for the study group fall below those for the other group. Thus, supplementation with n-3 fatty acids tended to improve duration of mechanical ventilation; however, this difference is not significant.

**Table 4:** **Clinical outcomes**

**Variable**	**Study group (** ***n*** **= 23)**	**Control group (** ***n*** **= 23)**	**Hazard ratio (95% CI)**	***p*** **value**
Duration of MV			1.663 (0.856–3.231)^a^	0.115
Mean (SE, 95% CI) days	13.61 (1.00, 11.66–15.56)	17.77 (1.81, 14.21–21.33)		
Median (SE, 95% CI) days	14 (2.38, 9.34–18.66)	17 (2.23, 12.64–21.37)		
Ventilator-free days^b^ (median, IQR)	14 (11–18)	11 (3–16)		0.172
Duration of ICU stay			2.182 (1.125–4.231)^a^	0.015
Mean (SE, 95% CI) days	17.6 3 (1.70, 14.30–20.97)	25.87 (2.6, 20.81–30.94)		
Median (SE, 95% CI) days	15 (3.19, 8.74–21.26)	24 (1.82, 20.44–27.56)		
ICU-free days^c^ (median, IQR)	13 (0–17)	4 (0–8)		0.028
Support free days^d^				
Renal replacement therapy, day	21 (0–24) (*n* = 7)	23 (14–25) (*n* = 7)		0.318
Catecholamine, day	26 (17.25–27) (*n* = 16)	26 (18–27) (*n* = 14)		0.728
Nosocomial infections, *n* (%)	10 (43.5)	12 (52.2)		0.768
Mortality, *n* (%)	3 (13.0)	3 (13.0)		1.000

*CI* confidence interval, *MV* mechanical ventilation, *SE* standard error, *IQR* interquartile range, *ICU* intensive care unit. ^a^The hazard ratio was calculated with the use of Cox proportional-hazards model; ^b^Ventilator free days were defined as the number of days between successful weaning from mechanical ventilation and day 28. ^c^ICU-free days were defined as the number of days between successful discharge from ICU and day 28; ^d^Support free days were defined as the number of days between the last day supported and day 28.

**Figure 3:**
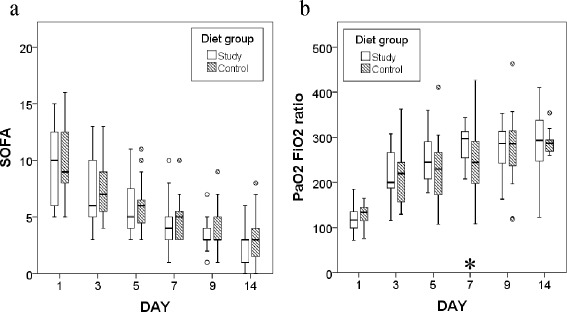
Box plots of changes in SOFA scores and PaO_2_/FiO_2_ over time. Circles represent data below 1.5 × IQR of the lower quartile or above 1.5 × IQR of the upper quartile. **(a)** SOFA scores. The number of patients for each day point is 23 for each group since the first censored case is at day 14. **(b)** PaO_2_/FiO_2_ ratios. The numbers of patients analyzed for each day point are the following: 23 vs. 23 (day 1), 23 vs. 23 (day 3), 23 vs. 23 (day 5), 23 vs. 22 (day 7), 18 vs. 21 (day 9), 12 vs. 14 (day 14), which is noted as “the study group” vs. “the control group”.

To evaluate the patients’ general conditions during the study, changes over time in SOFA scores and oxygenation status were assessed; these are plotted in Figure 3. Since all of the patients survived the first 2 weeks, there are no missing outcomes. SOFA scores decreased consistently in both groups; there was no significant difference at each time point. The PaO_2_/FiO_2_ ratio differed significantly only on day 7 (244.5 [197.5–295.8] vs. 297.0 [249.0–314.0], *p* = 0.021).

### Nutritional variables and adverse effects

Assessed nutritional variables and adverse events are shown in Table 5. For the first 7 days, the difference was not significant between the groups, except for serum concentrations of bilirubin (0.7 [0.5–1.0] vs 1.1 [0.8–2.4)], *p* = 0.009). Then, on day 14, the following three variables differed significantly: serum concentrations of creatine, triglyceride, and bilirubin. Neither vomiting nor abdominal distension occurred during the observation period. Six patients in the study group (21.6%) and four in the control group (17.4%) had mild, controllable diarrhea.

**Table 5:** **Hepatic and renal function**

	**Study group**			**Control group**		
	**Day 1**	**Day 7**	**Day 14**	**Day 1**	**Day 7**	**Day 14**
Platelets	88 (43–149)	172 (131–282)	273 (191–343)	95 (86–136)	214 (117–263)	224 (134–369)
AST (IU/L)	24 (16–32)	24 (19–36)	30 (20–52)	29 (18–38)	26 (21–43)	39 (23–51)
ALT (IU/L)	35 (23–48)	32 (23–47)	33 (23–44)	43 (29–57)	33 (19–44)	32 (23–50)
Creatine (mg/dL)^a^	0.96 (0.81–2.12)	0.54 (0.45–0.84)	0.48 (0.36–0.63)	0.92 (0.67–2.65)	0.68 (0.56–0.90)	0.60 (0.51–0.67)
Triglyceride (mg/dL)^b^	79 (62–103)	85 (72–98)	110 (94–131)	76 (55–98)	91 (62–119)	92 (79–123)
Cholesterol (mg/dL)	84 (76–105)	103 (80–120)	126 (95–142)	93 (76–143)	103 (83–121)	107 (87–126)
Bilirubin (mg/dL)^c^	1.0 (0.5–1.8)	0.7 (0.5–1.0)	0.8 (0.6–0.9)	1.5 (1.0–2.6)	1.1 (0.8–2.4)	1.0 (0.7–1.5)
Albumin (g/dL)	2.3 (2.1–2.7)	2.5 (2.2–2.8)	2.9 (2.5–3.2)	2.4 (2.2–2.9)	2.5 (2.2–2.8)	2.7 (2.2–3.1)

Values are expressed as medians (IQR). *AST* aspartate transaminase, *ALT* alanine transaminase. ^a^ *p* = 0.048 for day 14; ^b^ *p* = 0.035 for day 14; ^c^ *p* = 0.009 for day 7 and *p* = 0.028 for day 14.

## Discussion

In our study, supplementation with an enteral diet enriched with EPA, GLA, and antioxidants did not shorten the duration of mechanical ventilation, improve SOFA scores, or decrease the incidence of new nosocomial infections. From the statistical analysis, the power of this trial turned out to be very low for the primary outcome. It is very hard to statistically conclude whether the null hypothesis of no effect is actually true or not. From this analysis, we could design a further trial with 126 patients (63 per diet group) to detect 17.6% decrease of the duration of median mechanical ventilation (17 vs. 14) for a hazard ratio of 1.21 with a power of 80%.

The only significant improvements in the study group were in the duration of ICU stay and oxygenation status on day 7. Even the effects of the study diet on the clinical outcomes are minor; the effects to shorten ICU stay length is rather clinically relevant (median: 15 [standard error (SE): 3.19, 95% CI: 8.74–21.26] vs. median: 24 [SE: 1.82, 95% CI: 20.44–27.56]). The difference in median is 9 days, which is 37.5% reduction of the ICU stay. One rational of this improvement may be that tracheotomy was performed on more patients (13 cases) in the control group than those (8 cases) in the study group. Although it is not statistically significant, the length of the mechanical ventilation support for the control group was longer than that for the study group. As a result, more patients in the control group underwent tracheostomy because 2-week mechanical ventilation support is one of our criteria for tracheostomy (our two criteria are listed in Additional file 1). This difference could have caused the patients in the control group to stay longer in ICU. Further investigations are necessary to argue conclusively about the mechanisms of this benefit.

Regarding the significant difference of P/F ratio at day 7 between the study group and the control group, we assume that the anti-inflammatory effect of EPS, DHA, and antioxidants actually helped improving the oxygenation. Moreover, it is also known that n-6 fatty acid delays oxygenation improvement. These effects might lead to this significant distinction at day 7. The high variability of P/F ratio at day 7 in the control group is caused by only a few patients with relatively high oxygenation at day 7 in passing. One might argue that the oxygenation status improvement on day 7 also helped to shorten the ICU stay. We speculate, however, that this significant difference is rather tentative and does not directly contribute to the early discharge from ICU or the primary outcomes of the mechanical ventilation support duration. The medians of the weaning date are much later, day 14 of day 17. It is hard to believe that the difference on the day 7 solely affects the clinical outcomes 1 week later because that difference becomes statistically insignificant at day 9 and 14. Note that this speculation is not conclusive, and we are not able to deliver proper explanation of this distinction of P/F ratio at day 7. In contrast to our anticipation, enteral diets enriched with EPA, GLA, and antioxidants did not significantly improve the outcomes of sepsis-induced ARDS patients. We speculate that one possible reason for this result is the low protein intake at day 7 for both groups as shown in Table 1 (0.61 g/kg/day [0.53–0.68] vs. 0.78 g/kg/day [0.70–0.84]). Our enteral nutrition protocol specifies a low initial administration rate (70% of goal calories 1.2 × BEE in the first week). In case of acute disease, a deficit of energy supply (especially protein) in the first days could be crucial. On the other hand, we assume that this low initial administration rate might have contributed to our successful completion of enteral diets without discontinuation due to adverse effects such as uncontrollable gastrointestinal complications. Enteral nutrition provided at a minimum of 75% of goal calories for a minimum of 4 days reportedly confers significant benefits; however, adverse effects cause discontinuation of this regimen in some cases [11,13]. There is apparently an optimum rate that maximizes the benefits of the nutritional formula by conferring high efficacy and few and minor adverse effects.

Even though a systematic review concluded the beneficial effects of the supplementation with n-3 fatty acids [14], Grau-Carmona and colleagues [23] have demonstrated that the only benefit of an enteral diet enriched with these nutrients for critically ill patients with sepsis on mechanical ventilation is decreasing the duration of ICU stay. Their study is comparable with ours regarding study patients, study procedures, and clinical outcomes. As they discussed, the optimal dosage, timing, and nature of supplemental nutrients in patients with sepsis require clarification.

There are clinical trials reporting greater benefits with n-3 fatty acid and antioxidant-enriched enteral diets [11-13]. Although our findings do not support their strongly positive conclusions, we agree that n-3 fatty acid supplements have benefits for critically ill patients. Our argument concerns their choices of control diets. Our control group received a standard formula in which carbohydrates supply 54.5% of total calories; thus, n-6 fatty acids did not dominate our control diet. In contrast, in the studies cited above, fats supplied 55.2% of total calories and carbohydrates only 28.1% in both groups. The control diets in these studies were based on an enteral nutrition formula that is enriched with predominately n-6 fatty acids; these are metabolized into inflammatory eicosanoids [24,25]. Their study diets were enriched with n-3 fatty acids. The reported significant improvements in the study groups are possibly attributable to the absence of n-6 fatty acids in their diets. Moreover, their control groups may have had particularly unfavorable clinical outcomes. Even though our patients differed from those in the previous studies [11-13] in that they were older (70.2 years vs. 58.7 years) and had lower PaO_2_/FiO_2_ (122.4 vs. 179.0) with respect to mean values, higher mean SOFA scores (9.6 vs. 8.7), and more organ failures (2.09 vs. 1.45) as a mean value, the mortalities of the control groups in those three studies were much higher than in our control group (13.0% vs. 25–52%). We roughly estimated the mean values cited in the previous sentence by using the weighted means of the two groups in each trial. These findings suggest that the high concentrations of n-6 fatty acids administered to the control groups in the previous studies adversely affected the patients.

In the recently published OMEGA study of Rice and colleagues [26], enteral supplement with n-3 fatty acids, GLA, and antioxidants did not improve clinical outcomes or reduce inflammatory mediators in patients with acute lung injury. These researchers terminated the study prematurely because their first interim analysis suggested that receiving these nutrients was not merely futile but may even have been harmful. Although some of their results accord with ours, our findings suggest that enteral supplementation with n-3 fatty acids and antioxidants are safe. This apparent discrepancy might be attributable to their strategy of supplying n-3 fatty acids, GLA, and antioxidants by bolus feeding twice daily. In the OMEGA study, there were more cases of gastrointestinal intolerance manifested by diarrhea (28.7%) in the n-3 than in the control group; some patients possibly failed to absorb the supplemental nutrients adequately because of malabsorption. Malabsorption reportedly increases complications because of protein-energy malnutrition [27,28]. Therefore, supplying n-3 fatty acids in boluses may prejudice clarification of their effects.

Moreover, in our study we started nutrients within 24 h of a patient meeting the inclusion criteria, whereas in the OMEGA study, the starting time was up to 48 h after inclusion. Polymorphonuclear leukocyte infiltrates reportedly develop within 24 h when the lung is exposed to lipopolysaccharide [29]. In addition, a recently published study of patients in the early stages of sepsis without organ failure has demonstrated that early enteral feeding with EPA, GLA, and antioxidants decelerates progression to more severe sepsis and organ failures [30]. It is possible that early implementation of an enteral diet enriched with pharmaconutrients reduces deterioration of organ functions in patients with sepsis. Thus, the 24-hour difference between our study and the OMEGA study in starting nutrients might also have contributed to their differing conclusions.

We note that every patient registered is not admitted due to septic ARDS. Some patients are included in this study in the middle of their ICU stay since our inclusion time is defined to be the onset of septic ARDS. Excluding patients admitted due to septic ARDS, the duration from the ICU admission to the onset of the septic ARDS was 8.0 days (median, IQR: 6.5–13.0) in the study group and 5.0 days (median, IQR: 4.0–8.0). We believe that the ICU stay of this length before developing septic ARDS will cause little effect on the clinical outcomes once septic ARDS has been developed.

Our results may not be conclusive because we conducted our study in a single-center with a single-blind design, and the sample size was small. Nonetheless, our study still indicates that enteral nutrition enriched with n-3 fatty acid, GLA, and antioxidants has some benefits in critically ill patients. It is yet unclear which administration protocols most effectively maintain constant plasma concentrations of effective substances that are high enough to be beneficial. Further investigations are needed to clarify an optimum nutritional strategy with respect to administration route (enteral or parenteral or mixed), timing (bolus or continuous), rate of administration, start times, and the nutritional formula.

## Conclusions

This study showed that an enteral diet enriched with EPA, GLA, and antioxidants did not significantly improve duration of mechanical ventilation, total SOFA score, incidence of new nosocomial infections, and mortality in critically ill patients with sepsis-induced ARDS. However, we did demonstrate the benefits of significantly shorter duration of ICU stay and a trend toward moderately reducing duration of mechanical ventilation.

## Additional file

Additional file 1:
**Text 1.** Exclusion criteria; **Text 2.** Weaning protocol; **Text 3.** ICU discharge criteria; **Text 4.** Tracheotomy criteria; **Table S1.** Composition of the enteral diets.
