# Supplementary material for: Effect of enteral diet enriched with eicosapentaenoic acid, gamma-linolenic acid, and antioxidants in patients with sepsis-induced acute respiratory distress syndrome
Source: J Intensive Care. 2015 May 19;3(1):24. doi: 10.1186/s40560-015-0087-2 (PMC4443653; doi:10.1186/s40560-015-0087-2)
Supplement: Additional file 1: — Text 1. Exclusion criteria; Text 2. Weaning protocol; Text 3. ICU discharge criteria; Text 4. Tracheotomy criteria; Table S1. Composition of the enteral diets. [file 40560_2015_87_MOESM1_ESM.docx]

# **Supplementary Text 1. Exclusion criteria**

Exclusion criteria were age less than 18 years, receiving non-invasive mechanical ventilation, receiving short term mechanical ventilation (anticipated to be required for less than 72 hours), pregnancy or breast-feeding, enrollment in another trial within the previous 30 days, chronic malnourishment (defined as a body mass index [BMI] of < 17), BMI ≥ 30, hypertriglyceridemia (> 400 mg/dL), hypercholesteremia (> 240 mg/dL), inadequately controlled diabetes mellitus, chronic renal failure (glomerular filtration rate < 50 mL/min) or requiring dialysis, chronic liver disease of Child-Pugh stage C, heart failure of New York Heart Association grade IV, severe chronic respiratory failure under treatment, post-cardiopulmonary resuscitation, refractory shock, head trauma with Glasgow Coma Scale score ≤ 8, recent stroke or intracranial hemorrhage, neuromuscular disease, uncontrolled diarrhea, food allergy, recent gastrointestinal bleeding or ischemia, recent ileus, acute pancreatitis, malabsorption, short bowel syndrome, inflammatory bowel disease, extensive burns (≥ 40% of total body surface area), autoimmune disease, recent hematologic disease, HIV positivity, recent immunosuppressive therapy, neutropenia (absolute neutrophil count < 1500 cells/µL), and limited life expectancy (chronic or incurable disease such as terminal cancer).

# **Supplementary Text 2. Weaning protocol**

Here is our weaning protocol. The spontaneous breathing trial (SBT) is initiated if the PaO2/FIO2 ratio is greater than 200mm Hg on the condition of FIO2≦0.4 and PEEP≦8, hemodynamic status is stable without vasopressor support, and neuromuscular blocking agents are discontinued. The success criteria for SBT are following: SpO2≧90% and/or PaO2≧60 mmHg, spontaneous tidal volume≧4mL/kg, respiratory rate≦35, pH≧7.3, no respiratory distress, no sedations, satisfactory response to simple order. The attending physician decides whether to extubate (or change tracheostomy mask) if the patient tolerates the settings of CPAP≦5cm H2O with pressure support≦5cm H20 and FIO2≦0.5 for 60 minutes.

# **Supplementary Text 3. ICU discharge criteria**

Our discharge criteria from ICU is described as follows: stable respiratory state without ventilator support or high density oxygen in 12〜24 hour after the removal of the ventilator support, stable hemodynamics without hemodynamic support (e.g. vasopressor, fluid resuscitation, mechanical support), and no requirement of continuous renal replacement therapy. We confirm that, in this study, there was no patient whose discharge from ICU was extended even if those criteria were met.

# **Supplementary Text 4. Tracheotomy criteria**

There are two criteria for tracheotomy: 1) more than two weeks under the support of mechanical ventilation and 2) failures of the weaning more than once.

**Supplementary Table 1. Compositions of the enteral diets**

| **Nutrient** | **Study diet (per 100 mL)** | **Control diet (per 100 mL)** |
| --- | --- | --- |
| **Energy, kcal** | 150 | 100 |
| **Osmolarity, mOsm/L** | 384 | 330 |
| **Protein, g (%)** | 6.25 (16.7) | 3.52 (14.0) |
| **Sources** | 86.8% casein sodium | 59.6% casein sodium |
|  | 13.2% calcium caseinate | 27.3% calcium caseinate |
|  |  | 13.1% separation soy protein |
| **Carbohydrate, g (%)** | 10.6 (28.2) | 13.72 (54.5) |
| **Sources** | 45% maltodextrin | 71.4% dextrin |
|  | 55% sucrose | 28.6% sucrose |
| **Fat, g (%)** | 9.37 (55.1) | 3.52 (31.5) |
|  | 31.8% rape seed oil | 95.4% corn oil |
|  | 25% coconut oil | 0.46% soybean lecithin |
|  | 20% borage seed oil |  |
|  | 19.9% fish oil |  |
| **EPA, g** | 0.51 | 0 |
| **DHA, g** | 0.22 | 0 |
| **GLA, g** | 0.41 | 0 |
| **n-6 : n-3 ratio** | 1.6 : 1 | 44 : 1 |
| **Vitamins** |  |  |
| **palmitate, IU** | 528 | 250 |
| **β-Carotene , IU** | 672 | 0 |
| **Vitamin D3, IU** | 43 | 20 |
| **Vitamin E, mg** | 21.0 | 4.5 |
| **Vitamin C, mg** | 84.0 | 15.2 |
| **Folic acid, μg** | 42.0 | 20 |
| **Vitamin B1, mg** | 0.32 | 0.15 |
| **Vitamin B2, mg** | 0.36 | 0.17 |
| **Vitamin B6, mg** | 0.43 | 0.2 |
| **Vitamin B12, μg** | 0.6 | 0.6 |
| **Niacin, mg NE** | 2.9 | 2 |
| **Pantothenic acid, mg** | 1.3 | 0.5 |
| **Biotin, μg** | 6.0 | 15.2 |
| **Carnitine, mg** | 12.0 | 0 |
| **Minerals** |  |  |
| **Sodium, mg** | 131 | 80 |
| **Potassium, mg** | 196 | 148 |
| **Chloride, mg** | 169 | 136 |
| **Calcium, mg** | 106 | 52 |
| **Phosphorus, mg** | 100 | 52 |
| **Magnesium, mg** | 32 | 20 |
| **Iron, mg** | 2 | 0.9 |
| **Zinc, mg** | 2 | 1.5 |
| **Copper, μg** | 220 | 100.0 |
| **Selenium, μg** | 1.6 | 0 |
